# Factors Influencing the Attrition Rate of a 10-Week Multimodal Rehabilitation Program in Patients After Lung Transplant: A Neural Network Analysis

**DOI:** 10.3390/healthcare12222239

**Published:** 2024-11-10

**Authors:** Vanesa Dávalos-Yerovi, Dolores Sánchez-Rodríguez, Alba Gómez-Garrido, Patricia Launois, Marta Tejero-Sánchez, Vicenta Pujol-Blaya, Yulibeth G. Curbelo, Owen Donohoe, Ester Marco

**Affiliations:** 1 Rehabilitation Research Group, Hospital del Mar Medical Research Institute, 08003 Barcelona, Spain; doloresmaria.sanchez-rodriguez@chu-brugmann.be (D.S.-R.); emarco@psmar.cat (E.M.); 2 Physical Medicine and Rehabilitation Department, Hospital Vall d’Hebron, 08035 Barcelona, Spain; alba.gomezgarrido@vallhebron.cat (A.G.-G.); patricia.launois@vallhebron.cat (P.L.); vicenta.pujol@vallhebron.cat (V.P.-B.); 3 PhD Program in Biomedicine, Department of Experimental and Health Sciences, Universitat Pompeu Fabra—Doctoral School, 08002 Barcelona, Spain; 4 Geriatrics Department, Brugmann University Hospital, Université Libre de Bruxelles, 1020 Brussels, Belgium; 5 Geriatrics Department, Hospital Del Mar, Hospital de L’Esperança, Centre Fòrum, Parc de Salut Mar, 08029 Barcelona, Spain; 6 School of Medicine, Universitat Autònoma de Barcelona, 08193 Barcelona, Spain; 7 Physical Medicine and Rehabilitation Department, Hospital del Mar, 08003 Barcelona, Spain; mtejero@psmar.cat (M.T.-S.); yulibethgeraldine.curbelo.pena@psmar.cat (Y.G.C.); 8 Department of Parasitic and Infectious Diseases, Faculty of Veterinary Medicine, University of Liège, 4000 Liège, Belgium; owen.donohoe@uliege.be; 9 Bioscience Research Institute, Technological University of the Shannon, Athlone N37 HD68, Ireland; 10 School of Medicine, Universitat Pompeu Fabra, 08003 Barcelona, Spain

**Keywords:** attrition rate, compliance, lung transplant, artificial neural network, multimodal rehabilitation interventions

## Abstract

Background/Objectives: Despite the effectiveness of exercise and nutritional interventions to improve aerobic capacity and quality of life in lung transplant (LT) recipients, their compliance is low. Strategies to reduce the high attrition rate (participants lost over time) is a major challenge. Artificial neural networks (ANN) may assist in the early identification of patients with high risk of attrition. The main objective of this study is to evaluate the usefulness of ANNs to identify prognostic factors for high attrition rate of a 10-week rehabilitation program after a LT. Methods: This prospective observational study included first-time LT recipients over 18 years of age. The main outcome for each patient was the attrition rate, which was estimated by the amount of missing data accumulated during the study. Clinical variables including malnutrition, sarcopenia, and their individual components were assessed at baseline. An ANN and regression analysis were used to identify the factors determining a high attrition rate. Results: Of the 41 participants, 17 (41.4%) had a high rate of attrition in the rehabilitation program. Only 23 baseline variables had no missing data and were included in the analysis, from which a low age-dependent body mass index (BMI) was the most important conditioning factor for a high attrition rate (*p* = 7.08 × 10^−5^), followed by end-stage respiratory disease requiring PT (*p* = 0.000111), low health-related quality-of-life (HRQoL) (*p* = 0.0009078), and low handgrip strength (*p* = 0.023). Conclusions: The prevalence of high attrition rate in LT recipients is high. The profile of patients with a high probability of attrition includes those with chronic obstructive pulmonary disease, low BMI and handgrip strength, and reduced HRQoL.

## 1. Introduction

Lung transplant (LT) stands as the ultimate therapeutic option for patients with end-stage lung diseases because it not only enhances health outcomes but also extends the survival of carefully selected patients. The most prevalent conditions leading to LT are chronic obstructive pulmonary disease (COPD), cystic fibrosis (CF), idiopathic pulmonary fibrosis (IPF), and pulmonary arterial hypertension [1]. The number of LT has seen a significant increase in recent years. In 2022, Spain reached a notable milestone with 415 LTs, marking 33 years since the procedure’s inception in 1990. Similarly, France recorded 334 LTs [2] and the United States 2692 LTs [3]. This upward trend could be attributed to technical and medical advances, a growing pool of donors, and the expanded age limits for both donors and recipients, among other factors [1].

The American Thoracic Society/European Respiratory Society (ATS/ERS), among other international guidelines, emphasizes the importance of comprehensive respiratory rehabilitation programs [4,5,6]. These programs, incorporating exercise, nutrition, and other interventions such as psychological support and self-management strategies, are essential for improving aerobic capacity, quality of life, and long-term outcomes [7].

Despite this robust evidence, the implementation of exercise programs and nutritional care in clinical practice poses a significant challenge, as 40% of LT recipients referred to rehabilitation fail to complete the intervention [8]. Therefore, there is an urgent need for strategies to enhance compliance with these programs. The early identification of patients with a high probability of withdrawal could guide actions to increase compliance, and subsequently, improve clinical outcomes in this specific population. Currently, there is a lack of evidence on factors determining compliance with rehabilitation programs, probably due to the methodological challenge of predicting clinically relevant outcomes in small samples with a high attrition rate.

Artificial neural networks (ANNs) are analytical methods that use artificial intelligence and are designed to identify patterns while replicating intricate neural connections, mirroring the learning mechanisms of the human brain [9,10]. ANNs have been recognized for their utility in providing supportive information to clinical sciences, exploring factors that may impact the response to an intervention, identifying adjusting variables in a dataset, and developing prediction models.

Disease-related malnutrition with inflammation and sarcopenia are common in LT recipients, with rates reaching up to 80% for disease-related malnutrition [11,12] and 30–70% for sarcopenia [13,14]. Malnutrition leads to adverse consequences, including alterations in immune response, delayed wound healing, higher risk of sepsis, decreased diaphragm strength [15], higher mortality risk, and a higher risk of prolonged hospitalization [16]. Similarly, sarcopenia is associated with increased disability and hospitalizations, and decreased quality of life (QoL) in LT recipients [14,17,18]. The reversibility of both malnutrition and sarcopenia [19] makes early identification and targeted interventions particularly important.

We hypothesize that ANNs may aid in identifying factors influencing compliance with rehabilitation programs in this population. The primary objective of this study was to identify factors influencing the attrition rate of a 10-week multimodal rehabilitation program in patients after LT using an ANN model. Secondly, we aimed to assess whether malnutrition and sarcopenia, according to the most updated definitions [20,21], are associated with a higher attrition rate.

## 2. Materials and Methods

### 2.1. Study Design

This is a prospective observational study in LT recipients after a 10-week multimodal rehabilitation program. The Strengthening the Reporting of Observational Studies in Epidemiology (STROBE) guidelines were followed to report the research [22].

### 2.2. Setting

The study was conducted in a specialized outpatient clinics within the respiratory rehabilitation unit of a tertiary university hospital in Barcelona (Catalonia, Spain). This hospital serves as the sole reference center for LTs in Catalonia where the total population is 7.899.000 inhabitants. The study took place between January 2022 and September 2023.

Participants: This study included all first-time lung transplant (LT) recipients referred to the outpatient multimodal rehabilitation program as part of their standard LT care pathway. Participants adhered to the principles of rehabilitation and nutritional care as human rights [23,24]. Inclusion criteria included participants aged 18 years or older undergoing LT for the first time, being single or bilateral with a primary diagnosis of interstitial lung disease, COPD, cystic fibrosis, bronchiectasis or pulmonary vascular disease; within 3 months of discharge following LT; and able to speak and read Spanish or Catalan. Exclusion criteria included patients with pre-existing musculoskeletal disorders or conditions that could hinder study procedures; severe post-transplant critical illness neuromyopathy; and the presence of any other significant disease or disorder, which, in the opinion of the investigators, may put the participant at risk, influence the study results, or impede their ability to participate.

### 2.3. Multimodal Rehabilitation Program

The Multimodal Rehabilitation Program for LT recipients aims to enhance physical performance, aerobic capacity, nutritional status, and quality of life. The program follows a patient-centered process that includes assessing individual needs, setting goals, implementing therapeutic interventions, and conduction reassessments [25].

### 2.4. Nutritional Component

The nutritional assessment encompassed screening, diagnosis, monitoring, and a tailored care plan in accordance with ESPEN guidelines [26]. The screening involved validated scales, and the assessment covered phenotypic and etiologic indicators [27]. The patients followed a specialized dietary regimen characterized by hypercaloric and hyperproteic components during hospital stay to meet immediate post-transplant nutritional requirements set at 30–35 kcal/kg/day and 1.2 g/kg/day of proteins. Beyond the first month post-LT, nutritional targets transitioned to standard values applicable to the general population, specifically 25 kcal/kg/day and 0.8 g/kg/day of proteins. Education was provided to emphasize the importance of adhering to dietary guidelines and integrating lifestyle changes [26].

### 2.5. Exercise Component

Aerobic capacity was assessed by cardiopulmonary exercise testing (CPET), muscular strength was measured by 1-repetition maximum (RM) trough isometric dynamometry of peripheral muscles, and the measurement of maximal respiratory pressures. The intervention consisted of 20 supervised exercise sessions (1 h and 10 min sessions, 2 sessions per week, 10 weeks; total 21 h of training). Each session included 30 min of interval-based stationary bicycle exercise, 20 min of strength training, 10 min of breathing techniques, and 10 min of inspiratory muscle training.

- Aerobic training: Involved a 10 min warm-up phase, interval training on a cycloergometer (1 min of workload and 2 min of rest), and 10 min cool-down period. A structured enhancement in workload was introduced biweekly, starting from the minimum workload and progressing until reaching the maximum workload achieved during the CPET.
- Resistance training: Involved a supervised program, focused on strengthening major muscle groups. The resistance training was performed after the interval training and included 3 sets of 10 RM by leg press, arm press, back extension, and seated row using stationary machine weights. Frequency: Twice a week. Intensity: The principles of periodization and progression were applied [27]. Initially, the program used a load of 0.5 kg for upper limbs and 2 kg for lower limbs, based on individual tolerance. This load was progressively increased by 0.5 kg every two weeks, ensuring a challenging but safe workload for each participant. Time: Each resistance training session lasted approximately 20 min. Type: Resistance exercises were performed using free weights and elastic resistance bands. The targeted muscle groups included bicep curls, triceps extensions, core exercises, and leg presses.
- Inspiratory muscle training (IMT): IMT was customized based on the patient’s maximal inspiratory pressure (PImax), starting at 30% of the PImax. As the patient’s muscle strength improved, the resistance level was progressively increased by 10 cmH_2_O on a weekly basis using a respiratory trainer (Threshold IMT^®^, Philips Respironics, Chichester, UK). Breathing techniques, such as diaphragmatic breathing, controlled pursed-lip breathing, and segmental breathing, were also conducted for 10 min.

### 2.6. Variables

The primary outcome was the attrition rate. Studies into rehabilitation practices that involve participants recovering from serious illness often suffer from participant and/or data attrition. This is because health complications among participants can lead to some participants dropping out of the study, or make some measurements impractical, leading to incomplete or absent data, which poses challenges for analysis [28]. In this study, several relevant baseline measurements were taken from each participant at the start of a post-transplant rehabilitation program. A second measurement was later taken at the end in order to measure the impact of the program on several key patient related variables. Instances where measurements could not be taken due to participant dropout or unfavorable participant condition were recorded as missing data points, with the number of missing datapoints for each participant contributing to the overall attrition rate for each participant. At the end of the study, for each participant, the attrition rate was calculated based on the percentage of measurements that had missing data points. Subsequently, each participant was classified as having either a low attrition rate (<5% missing data), medium attrition rate (5–10% missing data), or high attrition rate (>10% missing data). At the end of the study, data for 23 common baseline variables that were complete (with no missing data points) for all participants were paired with the attrition rate classification for each participant and used as input/training data for the ANN (Supplementary Data S1).

Clinical variables, along with relevant data on exercise and muscle function collected prior to the multimodal rehabilitation program, were included in the ANN analysis as potential predictors or confounders of compliance. These variables encompassed the LT type (single or double LT) and indication (etiologic diagnosis), Charlson Comorbidity Index, body mass index (BMI), malnutrition screening, body composition parameters, peripheral and respiratory muscle strength, muscle thickness, physical performance, exercise tolerance, and quality of life. All variables were assessed both before and after the multimodal rehabilitation program.

### 2.7. Malnutrition

Malnutrition was assessed according to the Global Leadership Initiative on Malnutrition (GLIM) criteria, which involve three steps for assessment [20]. First, the risk was determined using both the Mini Nutritional Assessment Short Form (MNA-SF) [29] and the Malnutrition Universal Screening Tool (MUST) (range 0–6) [30]. The second step is diagnosis, involving at least one phenotypic and one etiologic criterion.

- Phenotypic criteria: (a) unintentional weight loss ≥5% in the last 6 months or ≥10% beyond 6 months; (b) low body mass index (kg/m^2^), defined as <20 kg/m^2^ or <22 kg/m^2^ in participants under and over 70 years of age, respectively; and c) fat-free mass index (FFMI) <17 kg/m^2^ in men and <15 kg/m^2^ in women using bioimpedance analysis (BIA) (101 Bodygram PLUS^®^ V.1.0 software) [31].
- Etiologic criteria: (a) reduced food intake defined as any gastrointestinal condition affecting food absorption or assimilation or reduction of ≤50% of energy requirements or any reduction sustained for more than 2 weeks; and (b) the presence of disease burden or inflammation. For study purposes, the criterion of disease burden was considered present in the entire sample of LT recipients.

### 2.8. Sarcopenia

The diagnosis of sarcopenia was defined as a loss of muscle mass and func-tion/strength (i.e., low fat-free mass and low handgrip strength), following the updated consensus of the European Working Group on Sarcopenia in Older People (EWGSOP2) [20]. Handgrip strength was assessed using a hand dynamometer (JAMAR^®^, Nottinghamshire, UK) and expressed in kilograms (kg). Patients performed a maximal voluntary isometric contraction of the finger flexor muscles, with the highest value among three reproducible maneuvers analyzed. Grip strength values were categorized as low based on the EWGSOP2 cutoff points (<27 kg in men, <16 kg in women) [21] and <80% of sex-specific Mediterranean normative values [32]. Muscle mass was assessed through BIA; appendicular skeletal muscle mass (ASMM) values <20 kg in men and <15 kg in women were considered as reduced [31]. The severity of sarcopenia was assessed by physical performance, measured by the Short Physical Performance Battery (SPPB) (range of 0–12), where ≤8 points indicated poor physical performance [33].

Quadricep strength was assessed using a digital dynamometer (MicroFet, Biometrics, Almere) and expressed in kg. The thickness of the dominant rectus femoris was measured by ultrasound (iU22, Phillips Medical Systems, Bothell, WA, USA) following the recommendations for the SARCopenia through UltraSound (SARCUS) group [34]. In the absence of validated cutoff points for the muscle specific strength, the 20th percentile was specifically calculated for the sample, and patients below the 20th percentile were considered to have a low muscle-specific strength, following the previously reported methodology [35].

### 2.9. Exercise Tolerance

Aerobic exercise capacity was estimated with the distance (m) travelled in a 6 min walking test (6 MWT) and the peak oxygen uptake (VO_2peak_) and the workload (W) achieved during a CPET through a standardized incremental cycle ramp protocol [36]. 

### 2.10. Other Pulmonary and Cardiovascular Function Tests

Maximal inspiratory and expiratory pressures (PImax and PEmax, respectively) were assessed by a pressure meter (MicroRPM™ Carefusion, Germany), following international guidelines [37]. The Modified Medical Research Council (mMRS) Dyspnea Scale was employed to assess the severity of dyspnea. This self-reported 4-stage tool ranges from 0 to 4, with higher scores indicating more severe dyspnea [38].

The patient evaluation included a broad range of variables, collected through a series of tests, to gain a comprehensive understanding of their health status and functional performance. Despite the number of assessments, the total duration of the initial evaluation was limited to 45 min. To optimize the process and minimize the need for unnecessary or duplicated tests, we used tests already requested by other specialists, such as the 6 min walk test (6 MWT), which were readily available in the patients’ medical records. This ensured that the evaluation was as efficient as possible, minimizing the burden on patients and ensuring that we obtained the most complete information possible.

### 2.11. Health-Related Quality of Life

Health-related quality of life (HRQoL) was evaluated using the EuroQoL-5D (EQ-5D) questionnaire, a standardized self-administered instrument consisting of a descriptive and a valuation section. The first part assesses five dimensions: mobility, self-care, usual activities, pain/discomfort, and anxiety/depression. Each dimension is rated as having no problems, some problems, or extreme problems. The second part features a visual analogue scale (EQ-5D VAS), represented as a thermometer with 100 intervals. A score of 100 indicates the best possible state of health, while a score of 0 represents the lowest conceivable state of health [39].

### 2.12. Ethics

The study was compliant with the ethical principles of the Helsinki Declaration, its further amendments (Fortaleza, 2013), and the Good Clinical Practice guidelines. Ethical approval was obtained from the Institutional Review Board of Hospital Vall d’Hebron (Nº PR AT530/2021). Informed consent was obtained from all participants. The data were gathered and treated in compliance with the current regulations in Spain and the General Data Protection Regulation (GDPR) established by the European Union in 2016/679 by the European Parliament and Council.

### 2.13. Statistical Methods

Categorical variables were represented using absolute numbers and percentages, while quantitative variables with the mean and standard deviation (SD) or the median and percentiles, depending on the nature of the data.

An ANN analysis was used identify the baseline variables that most influenced the attrition rate. Firstly, the training data (23 baseline variables and the primary outcome, which was the attrition rate) was prepared as described earlier. Next, this training dataset was prepared for the neural network. These data were utilized to train neural network using the “nnet” function in the nnet (v7.3.18) R package. After importing into R (v4.2.2), the variables were first defined as categorical ordinal, categorical, or numerical. Using R, attrition levels were encoded as 1, 2, and 3 for low, medium, and high, respectively. Using R core functions, numeric data were rescaled to between −1 and +1, for optimum compatibility with the “tanh” activator function used with the nnet R package. Ordinal categorical data were similarly rescaled, preserving ordinality and proportionality with 21 levels (0.1 increments) between −1 and +1 for each ordinal categorical variable. Using R core functions, scaled values from ordinal variables were rounded to one decimal place, to facilitate assignment to one of the 21 levels. 

The refined dataset was used to train the neural network, using attrition as the output/dependent variable, with all other remaining variables used as independent variables, with each assigned to an input neuron. Categorical variables were recoded using “dummy variables”, with each of these assigned to a separate input neuron to facilitate the processing of categorical data. The network had 30 neurons, a decay of 0.001, “tanh” activator, and linear output relationships. Convergence occurred at 3250 iterations. Garson’s algorithm assessed each input neuron’s contribution to attrition (on a scale of 0–1) using the R gar.fun function (downloaded from https://gist.github.com/fawda123/6206737, accessed on 10 August 2024). The resulting neural network model was visualized using the plot.net function in R (downloaded from https://gist.github.com/fawda123/7471137, accessed on 10 August 2024), with input neurons colored based on Garson’s algorithm outcomes.

Finally, regression analysis utilized the Garson’s algorithm outcome to identify variables with a contribution >0.5 to attrition. These selected variables were used in the regression analysis using the “polr” function from the MASS (v7.3.58.1) package in R, employing the logistic method option. The resulting model was analyzed with the “Anova” function from the car package (v3.1.1) in R for type-III ANOVA, thus determining each variable’s statistical significance in terms of their contribution to attrition rate classification. Plots were generated using ggplot2 (v3.4.3). Additional statistical comparisons between subpopulations were conducted using the Wilcoxon rank test via the “wilcox.test” function in R. Unless otherwise stated, all packages were downloaded directly from the Comprehensive R Archive Network (CRAN) using the install.packages function in R as per individual package installation instructions, all of which are available at https://www.rdocumentation.org/.

## 3. Results

Of the 41 patients referred to the multimodal rehabilitation program (mean age of 55.8 [SD 9.7] years; 22 men), 37 underwent double LT, with COPD being the most frequent indication for LT (29.3%). The mean time elapsed from LT to the initial assessment was 78.8 days (SD 43.9). Table 1 provides a description of the baseline characteristics of the study participants. All 33 (80.5%) patients categorized as at risk of malnutrition met the GLIM criteria, indicating a malnutrition prevalence of 80.5% (n = 33). The prevalence of sarcopenia was 56.1%, with 18 patients exhibiting an overlap of malnutrition and sarcopenia.

**Figure 1:**
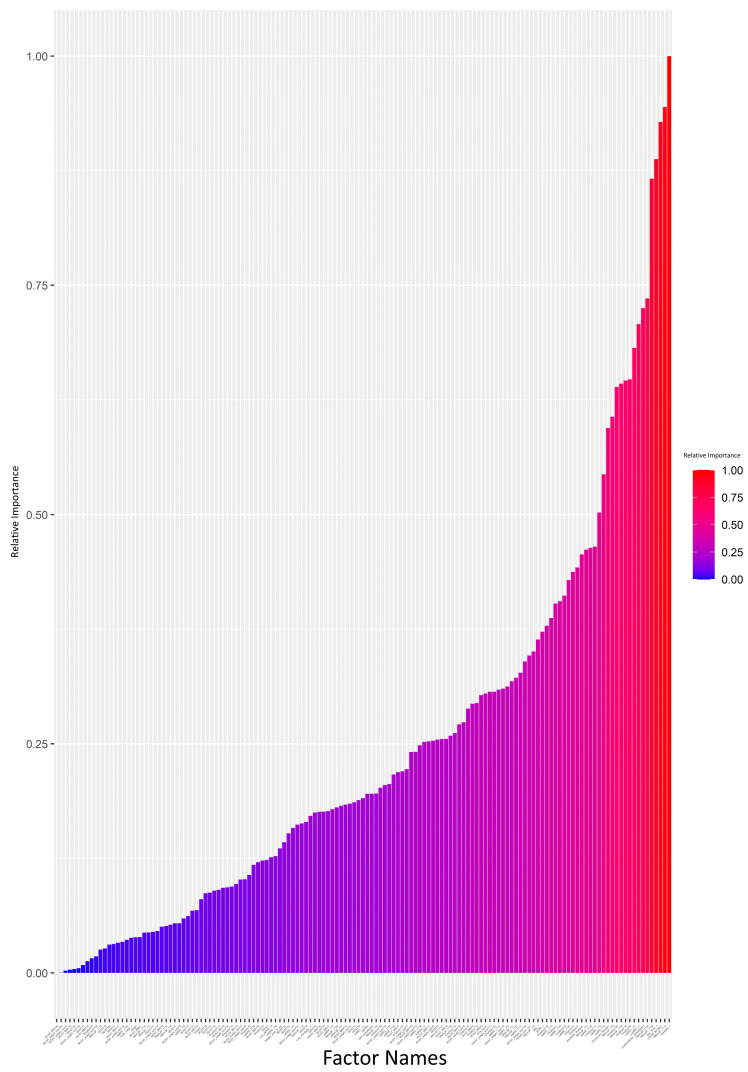
Impact analysis of variables on attrition: neural network model using the Garson’s algorithm (bar graph scale from 0 to 1). Each input variable was assigned a value in the range of 0–1 indicating its relative importance on impacting the attrition rate. The value for each input variable is also provided in Supplementary Data S2.

The attrition rate was high (>10%) in 17 (41.1%) patients, medium (5–10%) in 3 (7.3%), and low (<5%) in 21 (51.3%). The ANN model was generated and analyzed using a Garson’s algorithm to determine the relative contribution that each variable makes towards the levels of attrition. Each input variable was assigned a value from 0 to 1, indicating its relative importance in impacting the attrition rate. The relative importance of each input variable is visually summarized in Figure 1 and Supplementary Data S2. The ANN plot, annotated with the same relative importance values, is provided in Supplementary Data S1.

We set a 0.5 cutoff for relative importance values (Supplementary Data S2). Variables exceeding this cutoff, such as handgrip strength (kg), risk of malnutrition (MNA-SF), HRQoL, low handgrip strength according to EGWSOP2, dyspnea (mMRC), low age-dependent BMI according to GLIM, comorbidity (Charlson), and end-stage respiratory disease requiring PT, were deemed key contributors to attrition. The regression analysis focused on these variables. A broader regression analysis, incorporating all variables, showed limitations, failing to detect significant differences possibly due to overfitting or multicollinearity (Supplementary Data S3).

A refined regression model, which only included variables with relative importance > 0.5, indicated that a low age-dependent BMI (GLIM cutoff), low handgrip strength (EWGSOP2 cutoff and <80% Mediterranean reference values), HRQoL (EQ-5D VAS), and end-stage respiratory disease requiring PT all significantly contributed to data attrition (Table 2).

**Figure 2:**
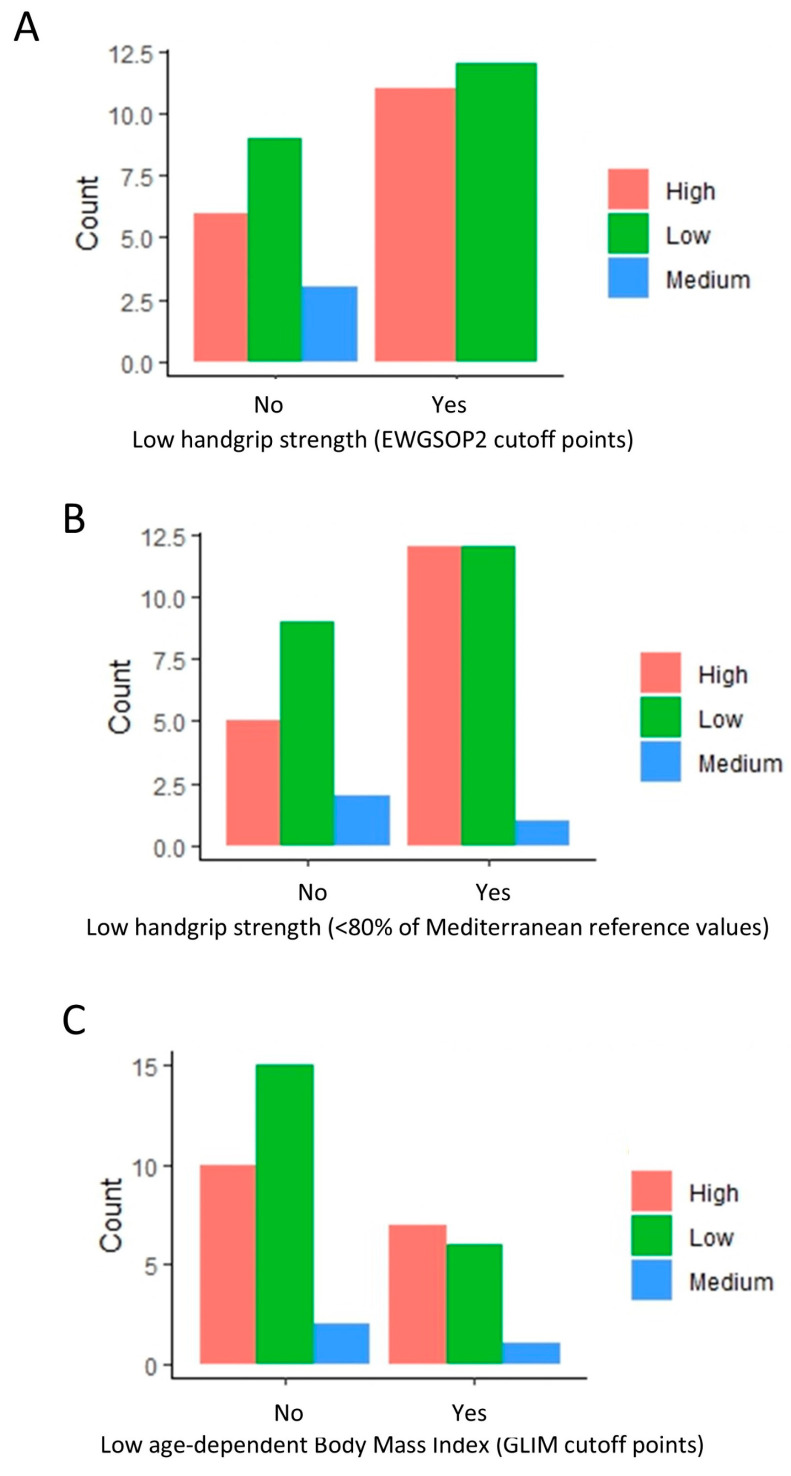
Distribution of patients with high, medium, and low attrition rates based on handgrip strength (EWGSOP and Mediterranean reference values) and body mass index. The bar plots indicate the amount of low, medium, and high attrition patients based on low handgrip (**A**,**B**) and low BMI (**C**) classification. A value of 0 indicates a normal handgrip strength and body mass index (BMI), and 1 indicates a low handgrip strength and BMI under the GLIM cutoff points.

Further scrutiny revealed a higher proportion of low attrition rates in patients without a low handgrip strength (EWGSOP) (Figure 2A). Interestingly, a comparable pattern persisted when using an alternate criterion for low handgrip strength (<80% of Mediterranean reference values) (Figure 2B). A similar trend was observed among patients without a low BMI (GLIM cutoff points) (Figure 2C), even when not included in the regression analysis.

**Figure 3:**
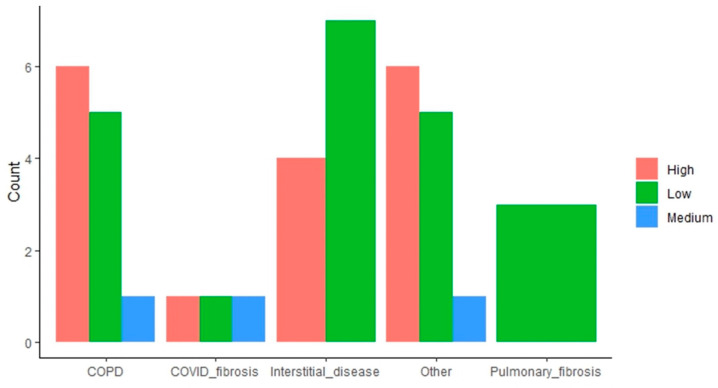
Distribution of patients with high, medium, and low attrition rates based on lung transplant indication. The bar plots indicate the amount of low, medium, and high attrition patients within each subpopulation based on the end-stage respiratory disease requiring LT.

The impact of the “end-stage respiratory disease requiring LT” variable is more mixed. Some indications (i.e., COPD, other indications) result in a higher proportion of high attrition patients, whereas interstitial disease and pulmonary fibrosis have the opposite effect, with these subpopulations having a higher proportion of low attrition patients (Figure 3).

**Figure 4:**
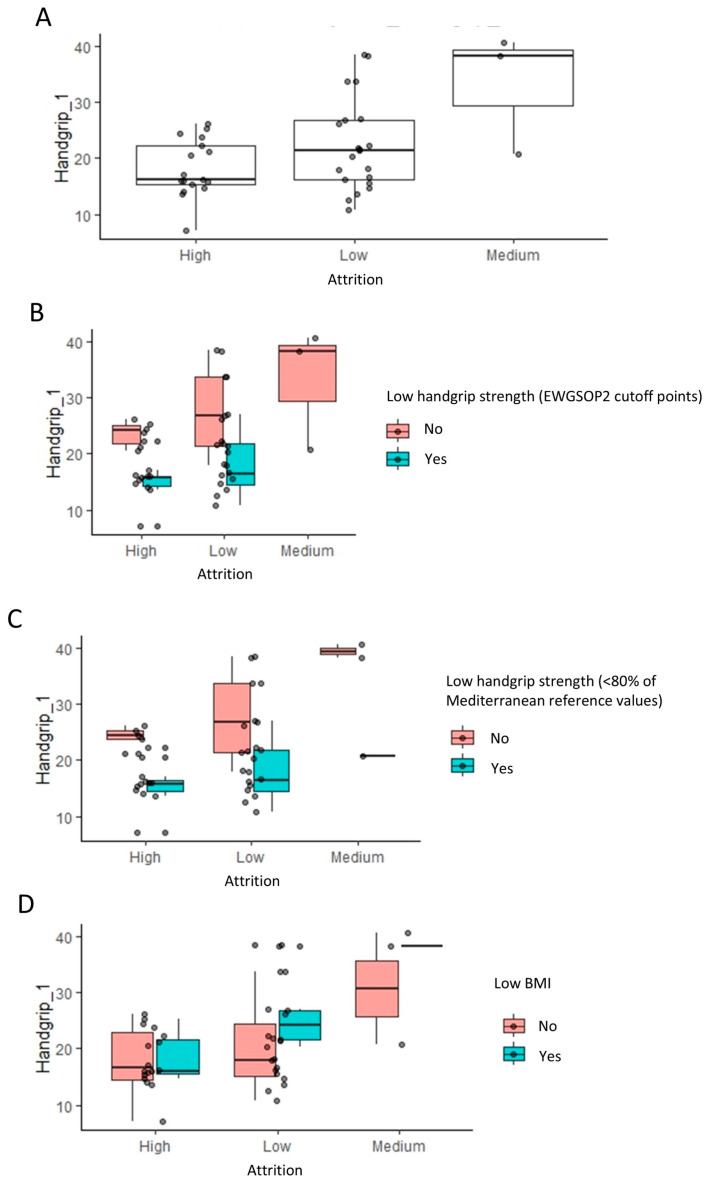
Impact of handgrip strength and body mass index on the attrition rates: linear regression analysis results. (**A**) The box plots indicate the handgrip strength levels across low, medium, and high attrition patients within each group for indication. To visualize the relationship to the categorical variables in Figure 2, these are further stratified by low handgrip and low BMI classifications (**B**–**D**).

Handgrip strength significantly influences attrition, evident in Figure 4A, where high attrition populations show a lower handgrip strength compared to low and medium attrition groups (*p* = 0.037, Wilcoxon rank test). Further stratification based on low handgrip strength (EWGSOP2) and low BMI classifications (Figure 4B–D) supports this, indicating that handgrip strength (kg) has a more substantial impact on attrition than categorized low handgrip strength (EWGSOP2). This observation is consistent with the fact that handgrip strength (EWGSOP2 cutoff point) was only under the significance threshold of 0.05 (Table 2). Despite a lower prevalence of high attrition in patients without a low BMI, they exhibited a slightly higher handgrip strength, suggesting a nuanced interaction among the variables influencing attrition.

**Figure 5:**
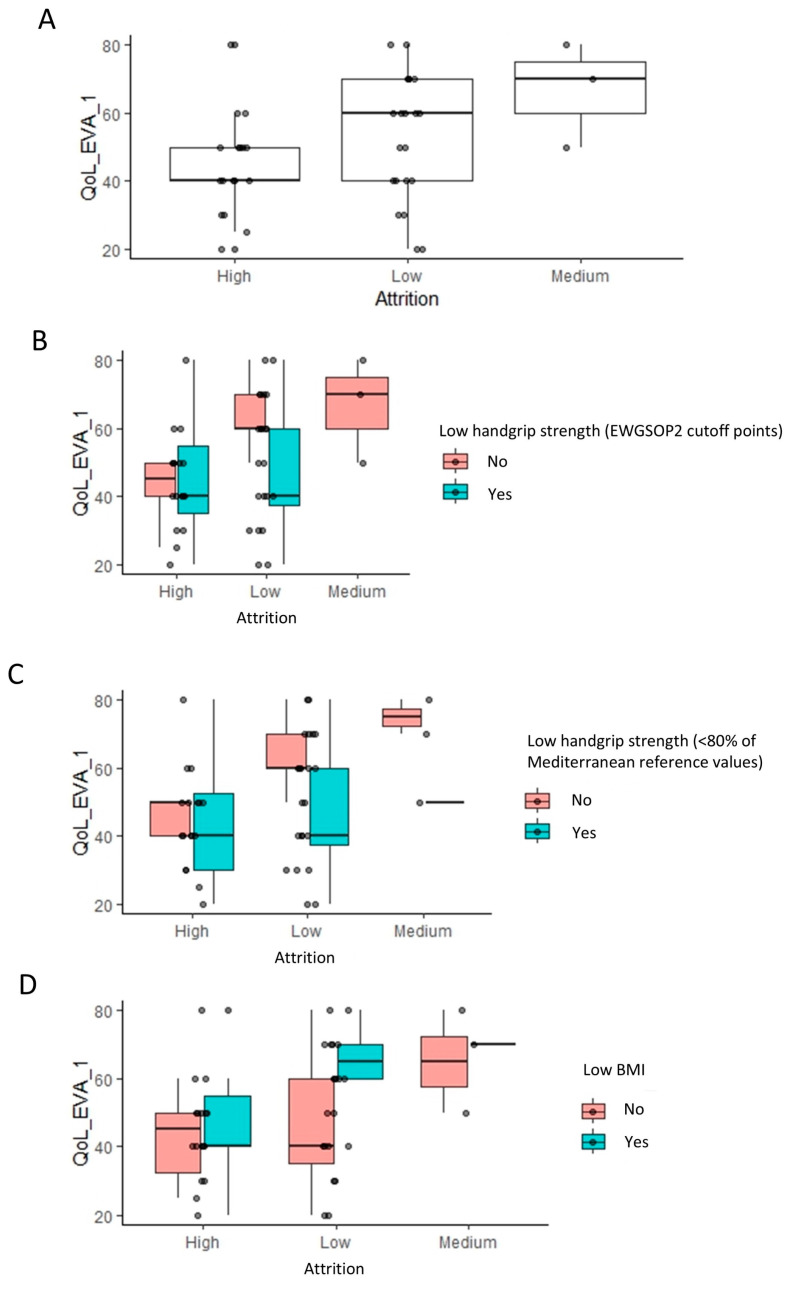
Impact of quality of life (EQ-5D) on the attrition rates: linear regression analysis results. Footnote: The linear regression analysis identified the visual analogue scale (VAS) of quality of life as having a significant impact on attrition (**A**). The box plots indicate the VAS of quality of life across low, medium, and high attrition patients. To visualize the relationship to the categorical variables in Figure 2, these are further stratified by low handgrip strength and low BMI classifications (**B**–**D**).

Notably, a very similar pattern was observed in between high, medium, and low attrition populations, when considering the impact of HRQoL. High attrition populations had a lower HRQoL score relative to low and medium attrition populations (*p* = 0.035, Wilcoxon rank test) (Figure 5A). Stratifying these populations based on the same three classifications as earlier revealed the exact same patterns that were observed earlier for the handgrip strength (Figure 5B–D).

## 4. Discussion

This study explored the factors conditioning the attrition rate of a multimodal rehabilitation program in LT recipients using an ANN model. The study identifies a low age-dependent BMI as the as the most important conditioning factor for a high attrition rate of the multimodal rehabilitation program (*p* = 7.08 × 10^−5^). Other important determinants include LT indication (*p* = 0.000111), HRQoL (*p* = 0.0009078), and handgrip strength (*p* = 0.023). The findings highlight the key role of these factors and suggest that patients with end-stage COPD, low HRQoL, low BMI, and handgrip strength represent the target population for interventions aimed at improving compliance.

This study is the first to assess the baseline factors associated with a high attrition rate in the framework of the new definitions of malnutrition and sarcopenia, including their individual components. Recent studies have delved into this critical aspect of rehabilitation programs [40,41], particularly among LT recipients. In these studies, the high attrition rate has been attributed to various factors, ranging from individual barriers to systemic issues within the healthcare framework. On an individual level, factors such as lack of motivation, competing life demands, or the perception of slow progress can lead participants to disengage from rehabilitation programs prematurely. Additionally, the presence of comorbidities, psychological factors, or socioeconomic constraints may further contribute to this phenomenon. Systemically, barriers such as limited access to rehabilitation services, inadequate social support, or program logistics can hinder sustained participation [41]. Tailored interventions, enhanced patient education, and ongoing communication between healthcare providers and patients can help improve engagement.

The very high prevalence of malnutrition and sarcopenia identified at baseline is in line with findings reported in LT recipients by other authors [11,12,13,14]. Lean body mass is significantly reduced in the pre- and post-transplant periods [42]. Since skeletal muscles are involved in all daily activities (walking, climbing stairs, dressing, bathing, etc.), a low muscle mass leads to physical activity intolerance and limitations in the activities of daily living [17]. The extent of the metabolic response triggered by the disease dictates the catabolic rate and the specific point during the disease’s progression at which malnutrition arises, along with its associated adverse outcomes [43]. The post-LT period remains complex, marked by pathophysiological changes impacting nutritional needs, which may also lead to malnutrition [44]. In the initial post-operative phase (from surgery to 6 months post-transplant), the body’s demand for nutrients escalates due to surgical wound healing. Simultaneously, nutritional support becomes crucial for meeting new metabolic requirements, replenishing energy stores, and ensuring the success of the graft. In the long-term phase (beyond 2 years post-transplant), numerous health concerns such as osteoporosis, malignancies, and multiple organ issues demand tailored nutritional interventions. Throughout these stages, the continuous use of immunosuppressive drugs and corticosteroids leads to both immediate and persistent adverse effects on nutrition, including altered appetite, taste changes, gastrointestinal symptoms, and metabolic imbalances [44].

In addition to the inherent limitations of a prospective observational study, several limitations should be acknowledged. First, the multimodal rehabilitation program focused solely on exercise and nutritional interventions, without including psychological or self-management components. Second, the high attrition rate observed throughout the process and during the initial analysis made it challenging to assess the impact of nutritional disorders, such as malnutrition and sarcopenia. Unfortunately, this attrition, combined with the limited sample size, compromised the ability to conduct a statistically meaningful analysis of the data related to these conditions. However, this limitation reflects transparency and the real-world challenge of clinical research. Third, this study did not investigate the influence of the rehabilitation program itself on participant attrition. This is a limitation, as the intervention’s role in influencing adherence is a critical aspect of rehabilitation programs. Future research could explore factors related to the intervention, such as perceived intensity, participant satisfaction, adequacy of the program to individual needs, or whether the intervention addressed specific needs and goals of the participants. We also explored the possibility of identifying predictive factors for responsiveness to the multimodal rehabilitation program, but the presence of missing data created a significant barrier and prevented imputations. However, most of the missing data were related to variables collected after the multimodal rehabilitation program. This makes sense, as data collection becomes more challenging afterward, either due to patient attrition or, even if the patient remains in the study, data collected from them may undergo a similar attrition process, with tests being limited to those that are least invasive. Given the complexity of the data, we explored the potential of combining neural network and regression analysis to identify baseline variables strongly associated with data attrition. In situations with many variables to consider, regression analysis with all variables is typically uninformative. Therefore, we aimed to use a neural network to identify the variables that contribute the most to attrition. While this could not directly allow us to infer statistical outcomes, it could help us to make logical, justified, and data-driven decisions regarding which variables to include in regression analysis. Thus, we first analyzed the dataset using a neural network and followed this with a targeted and rational design for regression analysis.

It is worth noting that the innovative aspects of the intervention, study design, and innovative analysis by ANN are the strong points of this research. The results derived from the ANN are relevant and can be translated to clinical scenarios within the context of a precision-based rehabilitation where strategies to improve compliance to rehabilitation intervention would be applied in specific cases.

## 5. Conclusions

The prevalence of high attrition rate was 41.7% in this sample of LT recipients after a multimodal rehabilitation program. The determinant factors conditioning this high attrition rate in the ANN were low age-dependent BMI, COPD, etiologic diagnosis, low HRQoL, and low handgrip strength. The requirement of including only the variables without missing data prevented us from determining the influence of malnutrition and sarcopenia according to the most updated definitions. However, future studies with new cohorts may facilitate the inclusion of these and other important variables in similar analyses to explore which baseline factors potentially contribute the most to attrition.

## Figures and Tables

**Table 1:** Baseline description of the participants (n = 41).

	Total Sample (n = 41)
Age (years), mean (SD)	55.8 (9.7)
Transplant type, n (%)	
-Double	37 (90.2)
-Single	4 (9.8%)
End-stage respiratory disease requiring LT, n (%)	
-COPD	12 (29.3)
-Interstitial disease	11 (26.8)
-COVID-19/Pulmonary fibrosis	6 (14.6)
-Other	12 (29.3)
Charlson Comorbidity Index, mean (SD)	2.7 (1.3)
Body mass index (kg/m^2^), mean (SD)	22.3 (4.5)
Handgrip strength (kg), mean (SD)	22.1 (8.2)
Quadricep strength (kg), mean (SD)	14.9 (7.2)
Quadricep thickness (mm), mean (SD)	10.6 (4.0)
Body composition parameters, mean (SD)	
Fat-free mass (kg)	
-Male	47.6 (4.4)
-Female	38.7 (6.8)
Phase angle (°)	
-Male	3.5 (0.9)
-Female	3.4 (0.7)
Appendicular skeletal muscle mass (kg)	
-Male	18.4 (2.2)
-Female	13.2 (3.5)
MUST, n (%)	
≥1 point	33 (80.5)
0 points	8 (19.5)
Malnutrition according to the GLIM criteria, n (%)	33 (80.5)
**Phenotypic criteria**	
-Unintentional weight loss	27 (65.9)
-Low body mass index	15 (36.6)
-Reduced muscle mass	29 (70.7)
**Etiologic criteria**	
-Reduced food intake or assimilation	2 (4.9)
-Disease burden/inflammation	41 (100)
Sarcopenia according to the EWGSOP2 criteria, n (%)	23 (56)
Short physical performance battery, mean (SD)	8.38 (2.9)
Six-minute walking distance (m), mean (SD)	345.7 (110.3)
Maximal inspiratory pressure (cmH2O), mean (SD)	59.8 (30.6)
Maximal expiratory pressure (cmH2O), mean (SD)	71.5 (24.9)
Maximal exercise load (W), mean (SD)	57.3 (19.4)
Oxygen consumption (mL/kg/min), mean (SD)	14.8 (3.7)
Oxygen minute volume (L/min), mean (SD)	42.9 (7.4)
Maximum heart rate (beats/min), mean (SD)	118 (16.1)
Oxygen pulse (mL/beat), mean (SD)	7.6 (1.8)
Quality of life (EQ-5D VAS), mean (SD)	50.1 (17.6)

Abbreviations: COPD: chronic obstructive pulmonary disease; EQ-5D: EuroQoL questionnaire 5 dimensions; EWGSOP2: European Working Group on Sarcopenia in Older People; GLIM: Global Leadership Initiative on Malnutrition; LT: lung transplant; MUST: Malnutrition Universal Screening Tool; SD: standard deviation; VAS: visual analogue scale; W: watts.

**Table 2:** Variables affecting the attrition rate: type-III ANOVA analysis and the regression model results.

Variable	*p*-Value
Low age-dependent body mass index (GLIM cutoff points)	**7.08 × 10^−5^**
End-stage respiratory disease requiring LT	**0.000111**
Quality of Life (EQ-5D VAS)	**0.0009078**
Low handgrip strength (<80% of Mediterranean reference values)	**0.0230351**
Low handgrip strength (EWGSOP2 cutoff points)	**0.0455796**
Modified Medical Research Council	0.3638802
Charlson Comorbidity Index	0.8804551

In bold, statistically significant variables. Abbreviations: EQ-5D: EuroQoL questionnaire 5 dimensions; EWGSOP2: European Working Group on Sarcopenia in Older People; GLIM: Global Leadership Initiative on Malnutrition; LT: lung transplant, VAS: visual analogue scale.

## Data Availability

The original contributions presented in the study are included in the article/Supplementary Materials; further inquiries can be directed to the corresponding author.

## Supplementary Materials

The following supporting information can be downloaded at: https://www.mdpi.com/article/10.3390/healthcare12222239/s1, Supplementary Data S1: Data used as input for ANN, including 23 common baseline variables that exhibited no missing data points with all participants, and the corresponding attrition rate classification for each participant. Attrition rate for each participant was calculated based on the amount of missing data across all variables (i.e., percentage of missing data per participant across all baseline/pre-rehabilitation and post-rehabilitation measurements). Subsequently, each participant was classified as having either a low attrition rate (<5% missing data), medium attrition rate (5–10% missing data), or high attrition rate (>10% missing data). Supplementary Data S2: Results from analysis of the neural network model using the Garson’s algorithm. Each input variable was assigned a value from 0-1 indicating its relative importance in impacting the attrition rate. The higher the value, the more importance a variable has on attrition rates. Variables with relative importance >0.5 were defined as being among the most important and were selected for regression analysis and are highlighted in bold. Categorical variables were highlighted in bold if at least one level was >0.5. * Note: As part of this process, the levels of each categorical and ordinal categorical variable were automatically converted into new individual variables and renamed by R. Variables representing rescaled levels of each ordinal-categorical variable were given the ending “^” followed by a number or letter. Variables representing levels of each categorical variable were followed by a number. Supplementary Data S3: *p*-values from type-III ANOVA analysis on the regression model using all variables. No Significance was observed for any variable using this model possible due to overfitting and/or multicollinearity.
